# Supplementary material for: Qualitative study: patients’ enduring concerns about discussing internet use in general practice consultations
Source: BMJ Open. 2021 Apr 28;11(4):e047508. doi: 10.1136/bmjopen-2020-047508 (PMC8094327; doi:10.1136/bmjopen-2020-047508)
Supplement: Supplementary data [file bmjopen-2020-047508supp001.pdf]

SUPPLEMENTAL MATERIAL 1 – PATIENT CONSENT FORM

Title of Project: **Harnessing resources to maximise outcomes from GP consultations (HaRI): a qualitative study**

Please initial all boxes

1. I confirm that I have read and understand the information sheet provided dated 15th July 2016 (v 1.2) for the above study. I have had the opportunity to consider the information, ask questions and have had these answered satisfactorily. ☐

2. I understand that my participation is voluntary and that I am free to withdraw at any time up until the end of data collection at this site without giving any reason. ☐

3. I understand that my interview will be audio-recorded. ☐

4. I understand that the interview will be transcribed by a professional transcribing company with details anonymised to protect my identity. ☐

5. I agree to the use of anonoymised verbatim quotation for the purposes of education and teaching (for example publication, conference presentations). ☐

CONSENT TO POINTS 6 AND 7 IS OPTIONAL AND NOT REQUIRED FOR STUDY PARTICIPATION

6. I consent for the transcript of the interview to be placed into an archive at UCL to allow data to be used, subject to an appropriate protocol and ethical approval, by other researchers. I understand this carries a potential risk that I might be identified. ☐

7. I consent for the audio tape to be placed into an archive at UCL to allow data to be used, subject to an appropriate protocol and ethical approval, by other researchers. I understand this carries a potential risk that I might be identified. ☐

Name of Participant	Date (DD/MM/YY)	Signature
Name of person taking consent	Date (DD/MM/YY)	Signature

SUPPLEMENTAL MATERIAL 2 – INTERVIEW TOPIC GUIDE

Interview Topic Guide: Patients

UCL

Topic Guide : Interviews with patients

Harnessing resources from the internet to maximise outcomes from GP consultations (HaRI): a qualitative study

1. **Consultation**

Could you start by describing what you can remember about the consultation we videorecorded.

2. **Sources of information and advice**

Before your appointment with [GP's name], did you seek information or advice about symptoms, conditions, treatments or health services?

INTERNET MENTIONED

Focusing on the information/advice you looked for on the internet, can you remember what information/advice you were looking for?

Did you refer to the information/advice you looked for on the internet during your consultation with [GP's name]?

-Yes

How did the doctor respond?

-No

Was there any reason why you did not refer to the information/advice you looked for on the internet during your consultation?

NO MENTION OF INTERNET

Did you consult the internet for information or advice about symptoms, conditions, treatments or health services before your appointment with [GP's name]?

-Yes

Did you refer to this during your consultation?

-Yes

How did the doctor respond?

-No

Was there any reason why you did not refer to the information/advice you looked for on the internet during your consultation?

……………………………………………………………………………………………..

-No

Have you looked for information or advice about symptoms, conditions, treatments or health services on the internet before your consultation with the doctor on some other occasion?

YES

Did you refer to this during your consultation?

-Yes

How did the doctor respond?

-No

Was there any reason why you did not refer to the information or advice you looked for on the internet during your consultation?

3. GP use of internet for diagnosis/treatment recommendation

Has the doctor ever looked something up on the internet during your consultation?

-Condition

-Treatment

YES

-Can you remember any particular occasions?

-What were your thoughts on the doctor doing that?

NO

-What do you think about doctors looking up information on the internet during consultations?

4. GP use of internet for explaining condition/treatment

Has the doctor ever used information from the internet to help him/her explain a condition or treatment?

-Talked through information on a webpage

-Images

YES
-Can you remember any particular occasions when your doctor has done this?
-What were your thoughts on the doctor doing that?

NO
What do you think about doctors using information from the internet to help explain a condition or treatment during consultations?

5. GP printing out information from the internet for patient to take away

Has the doctor ever printed out information from the internet about a health condition or treatment for you to take away and read?

YES
-Can you remember any particular occasions when your doctor has done this?
-What were your thoughts on the doctor doing that?

NO
What do you think about doctors printing out information from the internet for patients to take away and read?

6. GP recommendation

Has the doctor ever recommended a particular website during your consultation?

YES
-Can you remember any particular occasions when your doctor has done this?
-What were your thoughts on the doctor doing that?

7. GP warning

Has the doctor ever suggested that you don't look for information or advice on the internet?

YES
-What did you think about the doctor doing that?

**NO**
-What do you think about doctors suggesting to patients that they should not look for health information or advice on the internet?

8. Anything to add

Is there anything you would like to add about:
-The information patients and GPs use in and outside of consultations to manage health problems
-GP consultations more generally

9. Reflections on taking part in the research

How did you find participating in the study?
