# Supplementary material for: Qualitative study: patients’ enduring concerns about discussing internet use in general practice consultations
Source: BMJ Open. 2021 Apr 28;11(4):e047508. doi: 10.1136/bmjopen-2020-047508 (PMC8094327; doi:10.1136/bmjopen-2020-047508)
Supplement: Author's manuscript [file bmjopen-2020-047508.draft_revisions.pdf]

BMJ Open is committed to open peer review. As part of this commitment we make the peer review history of every article we publish publicly available.

When an article is published we post the peer reviewers' comments and the authors' responses online. We also post the versions of the paper that were used during peer review. These are the versions that the peer review comments apply to.

The versions of the paper that follow are the versions that were submitted during the peer review process. They are not the versions of record or the final published versions. They should not be cited or distributed as the published version of this manuscript.

BMJ Open is an open access journal and the full, final, typeset and author-corrected version of record of the manuscript is available on our site with no access controls, subscription charges or pay-per-view fees (http://bmjopen.bmj.com).

If you have any questions on BMJ Open's open peer review process please email info.bmjopen@bmj.com

BMJ Open

Qualitative Study: Patients' Enduring Concerns about Discussing Internet Use in General Practice Consultations

Journal:	*BMJ Open*
Manuscript ID	bmjopen-2020-047508
Article Type:	Original research
Date Submitted by the Author:	05-Dec-2020

Complete List of Authors:	Cuteanu, Anita; University College London Research Department of Primary Care and Population Health,<br>Seguin, Maureen ; London School of Hygiene and Tropical Medicine<br>Ziebland, Sue; University of Oxford, NDPCHS<br>Pope, Catherine; University of Oxford, Nuffield Department of Primary Care Health Sciences<br>Leydon, Geraldine; University of Southampton, Faculty of Medicine<br>Barnes, Rebecca; Bristol University, Centre for Academic Primary Care, School for Social and Community Medicine<br>Murray, Elizabeth; University College London Research Department of Primary Care and Population Health<br>Atherton, Helen; University of Warwick,<br>Stevenson, Fiona; University College London Research Department of Primary Care and Population Health

Keywords:	PRIMARY CARE, QUALITATIVE RESEARCH, World Wide Web technology < BIOTECHNOLOGY & BIOINFORMATICS

SCHOLARONE™
Manuscripts

I, the Submitting Author has the right to grant and does grant on behalf of all authors of the Work (as defined in the below author licence), an exclusive licence and/or a non-exclusive licence for contributions from authors who are: i) UK Crown employees; ii) where BMJ has agreed a CC-BY licence shall apply, and/or iii) in accordance with the terms applicable for US Federal Government officers or employees acting as part of their official duties; on a worldwide, perpetual, irrevocable, royalty-free basis to BMJ Publishing Group Ltd ("BMJ") its licensees and where the relevant Journal is co-owned by BMJ to the co-owners of the Journal, to publish the Work in this journal and any other BMJ products and to exploit all rights, as set out in our licence.

The Submitting Author accepts and understands that any supply made under these terms is made by BMJ to the Submitting Author unless you are acting as an employee on behalf of your employer or a postgraduate student of an affiliated institution which is paying any applicable article publishing charge ("APC") for Open Access articles. Where the Submitting Author wishes to make the Work available on an Open Access basis (and intends to pay the relevant APC), the terms of reuse of such Open Access shall be governed by a Creative Commons licence – details of these licences and which Creative Commons licence will apply to this Work are set out in our licence referred to above.

Other than as permitted in any relevant BMJ Author's Self Archiving Policies, I confirm this Work has not been accepted for publication elsewhere, is not being considered for publication elsewhere and does not duplicate material already published. I confirm all authors consent to publication of this Work and authorise the granting of this licence.

For peer review only

Main Document

Title: Qualitative Study: Patients' Enduring Concerns about Discussing Internet Use in General Practice Consultations

Authors: Cuteanu Anita, Seguin Maureen, Ziebland Sue, Pope Catherine, Leydon Geraldine, Barnes Rebecca, Murray Elizabeth, Atherton Helen, Stevenson Fiona.

1- Ms Anita Cuteanu
(Corresponding Author)
a.cuteanu@ucl.ac.uk
1. Laidlaw Fellow,
University College London Research Department of Primary Care and Population Health
Gower St, Bloomsbury
London, London, UK NW3 2PF

2- Dr Maureen Seguin, BA, MA, PhD
Maureen.Seguin@lshtm.ac.uk
1. Research Fellow,
London School of Hygiene & Tropical Medicine
London, London, UK WC1E 7HT

3- Professor Sue Ziebland, BA, MSc, PhD
sue.ziebland@phc.ox.ac.uk
1. Professor of Medical Sociology,
University of Oxford Nuffield Department of Primary Care Health Sciences
Oxford, Oxfordshire, UK OX3 7LF

4- Professor Catherine Pope, BA (Hons), PhD
catherine.pope@phc.ox.ac.uk
1. Professor of Medical Sociology
University of Oxford, Nuffield Department of Primary Care Health Sciences
Oxford, Oxfordshire, UK OX1 2JD

5- Professor Geraldine Leydon, BA, MA, PhD, Dip. PH
G.M.Leydon@soton.ac.uk
1. University of Southampton
Southampton, Hampshire, UK SO17 1BJ

6- Dr Rebecca Kate Barnes, BSc(Hons), PGDip., PhD
Rebecca.Barnes@bristol.ac.uk
0000-0001-8844-7496
1. Senior Research Fellow
University of Bristol, Bristol Medical School, Population Health Sciences
Canynge Hall

For peer review only - http://bmjopen.bmj.com/site/about/guidelines.xhtml

39 Whatley Rd
Bristol, UK BS8 2PS

7- Professor Elizabeth Murray, FRCGP FRCP(Edin) PhD
elizabeth.murray@ucl.ac.uk
1. Professor of e-Health and Primary Care
University College London Research Department of Primary Care and Population Health
London, London, UK NW3 2PF

8- Dr Helen Atherton, BSc, MSc, PhD
h.atherton@warwick.ac.uk
0000-0002-7072-1925
1. Associate Professor
University of Warwick, Warwick Medical School
Gibbet Hill Road
Coventry, UK CV4 7AL

9- Professor Fiona Stevenson, MA (Hons), PgDip., PhD
f.stevenson@ucl.ac.uk
1. Professor of Medical Sociology,
University College London Research Department of Primary Care and Population Health
Gower St, Bloomsbury
London, London, UK NW3 2PF

Word count: 3232 words.

For peer review only

ABSTRACT:

Objectives: To examine patients' accounts of their use of the internet before seeing a general practitioner (GP) using thematic analysis of semi-structured interviews.

Setting: Primary care patients consulting with 10GPs working at 7 GP practices of varying sizes and at a range of locations around London and the surrounding Southeast area of England.

Participants: 28 adult patients: 14 male patients and 16 female patients of ages ranging from 18 to 75 and belonging to varying ethnic groups. They were selected based on instances when the patients themselves reported having used the internet before or during the consultation or when the physician used the internet or made reference to it during the consultation.

Results: Patients report that they can find reliable health information online which they believe is helpful for both themselves and their GP. However, they report uncertainty about how to share internet-based findings and reluctance to disclose their efforts at researching health issues online for fear of appearing disrespectful or interfering with the flow of the consultation.

Conclusions: Despite the democratisation of access to information about health due to the internet patients continue to experience their use of the internet for health information as a sensitive and potentially problematic topic. The onus may well be on GPs to raise the likelihood (without judgement) that patients will have looked things up before consulting and invite them to talk about what they found.

STRENGTHS AND LIMITATIONS OF THIS STUDY

- This study serves to strengthen our findings in relation to patients' enduring concerns about discussing their internet use in general practice consultations.
- Only patients who agreed to have their consultation video recorded for the main study ('Harnessing resources from the internet to maximise outcomes from GP consultations' (HaRI)) were invited to take part in the interview study.
- Of those, only patients for whom the internet was raised in either their pre-consultation survey, or their consultation, were approached.

INTRODUCTION:

The internet, the "global computer network" [1] connecting and providing us with unlimited information at our fingertips, has become increasingly present in everyday life, with daily use having more than doubled in the last twelve years in the U.K. [2, 3] It is not surprising that its use for health-related information has followed the same trend, [2] especially as British health authorities and successive governments have increasingly encouraged the population to become more proactive and independent regarding their own healthcare and health management. [3-6] In summary, the internet has become an important source of a wide range of medical information which is easily accessible by those without medical training. [2, 7]. Indeed, Professor Helen Stokes-Lampard, former Chair of the Royal College of General Practitioners highlighted the

For peer review only - http://bmjopen.bmj.com/site/about/guidelines.xhtml

increasing pervasiveness of the internet in consultations, dubbing its presence as that of "Dr. Google". [8]

However, while patients are being encouraged to take on more responsibility and play a greater role in their own health care, both GPs and patients are concerned about how discussions of internet-based health information affect relationships in the consultation. [9,10] Patients report a desire to maximise the available time in consultations by preparing in advance and informing themselves of problems and possible solutions. [9] Nevertheless, they remain mindful of not wanting to appear to challenge their GP's expertise. [9] Conversely, GPs express anxiety about the potential for their status and expertise to be called into question. [10] In some cases, patients may even find themselves trapped, to at least some degree, in what Bloor and Hororbin described as a "double-bind" which expects them to be sufficiently informed, but still defer to their GP's medical expertise and feign ignorance in relation to their medical problem. [11]

This paper offers an in-depth examination of patients' accounts concerning their use of the internet before, during and following GP consultations. Given the aforementioned persistent unease surrounding the use of the internet in the consultation room, we reflect on the ways in which patients discuss and account for their use of the internet with regard to general practice consultations.

METHODS

Design

Qualitative interview study as part of the Harnessing Resources from the Internet (HaRI) study. [12] The complete data set comprises 281 video-recorded general practice (GP) consultations, with pre-consultation questionnaires completed by all patients, interviews with all 10 participating doctors and 28 selected patients. The 10 GPs came from seven practices across London and the southeast of England. Practices varied in terms of size, population density, level of deprivation and whether they were a training practice.

Data

We focus on the 28 patients who were interviewed after the consultation. Data were collected between September 2017 and May 2018. Ethical Approval was obtained from a local UK NHS Research Ethics Committee, and governance approval from the Health Research Authority.

Data collection

Two to three patients were purposively selected (on the basis of survey and consultation data) from each practice. Patients were selected to reflect (i) instances in which patients reported searching the internet before their GP consultation and this was raised in the consultation, (ii) instances in which patients reported searching the internet before their GP consultation and this was NOT raised in the consultation, (iii) instances in which the patient raised the topic of the internet in the consultation and this was not reported in the pre-consultation questionnaire and (iv) instances in which the GP used the internet or raised it as a topic during the consultation.

We sought to provide maximum variation in terms of socio-demographic characteristics. All participants provided signed and informed consent which included permission to publish anonymised extracts for the purpose of education and teaching. It was made clear that the decision

For peer review only

to participate or to decline would not affect clinical care and that it was possible to withdraw consent up to the time all data were collected at their site.

Interviews were conducted by one of two experienced female researchers. They took place either in patients' homes or, if preferred, in a room provided by their GP practice. Semi-structured interviews were conducted by two experienced, non-clinical, qualitative researchers. Interviews varied in length from 25 to 90 minutes and were audio recorded. All followed an agreed topic guide (See Supplemental Material 1), which focused on accounts of what patients did prior to consulting, with prompts relating to the internet if it was not raised. Patients were also asked what happened in the consultation. The interviews were transcribed verbatim and anonymised by a professional transcription service with the transcripts checked for accuracy by our team.

Data analysis

The data were analysed using thematic analysis [13, 15, 16] Data analysis began with the process of familiarisation, noting down general impressions and providing a content summary for each interview. Initial codes and impressions were carefully sorted and collated into overarching themes. [14] Employing, as Nowell et al. [14] put it, "an iterative and reflective process that develops over time and involves a constant moving back and forward between phases" [14, p.4] A series of coherent themes were developed, reviewed and defined over a period of several weeks. A data clinic session was held with seven experienced qualitative researchers to help further refine and strengthen the existing themes. Additional discussions and brainstorming sessions with the project leader (FS) helped structure the themes and develop the narrative.

FINDINGS

Participant characteristics:

Interviewees comprised 14 female and 14 male patients ranging in age from 18 to 75 years of age (see Table 1).

Table 1: Characteristics of patients interviewed

Gender	Age	Condition
M	56 - 65	Foot problem
F	56 - 65	Urinary tract infection
M	56 - 65	Suspected inner-ear benign tumour.
F	56 - 65	Blood in stools
F	19 - 25	Throat feel bruised
F	56 - 65	Blood test result
M	46 - 55	Knee pain.
F	46 - 55	Shoulder pain
M	56 - 65	Suspected hernia.
F	66 - 75	Requested knee replacement.

For peer review only - http://bmjopen.bmj.com/site/about/guidelines.xhtml

M	66 - 75	Hole where hernia operation scar is.
M	66 - 75	PSA test result
F	36 - 55	Thyroid problem
F	66 - 75	Hissing sound in one ear
F	26 - 35	Pain in ear.
F	26 - 35	Complications from deviated septum surgery
M	0 -18	Baby has oozing belly button
F	66 - 75	Respiratory infection
F	66 - 75	UTI and Heart issues
F	56 - 65	Pain behind knee.
M	0 -18	Three-year-old boy has spot on neck.
M	46 - 55	Discussion of patient's recovery from throat cancer.
F	56 - 65	Review of patient's overactive thyroid.
M	46 - 55	Lethargy, pain in chest.
M	56 - 65	Check up for diabetes and.
M	66 - 75	Review of pain in head.
F	46 - 55	Lower back pain, numbness in tip of middle finger.
F	46 - 55	Mark on chest

Internet Research – A Complicated but Necessary First Step for Patients?
Although the literature suggests that patients are worried about being perceived negatively, in this study most patients presented consulting the internet as an important and necessary step prior to seeing their GP. [2] Researching symptoms or concerns on the internet via the well-known web search engine "Google" has become a reflex for many. This is a testament to its perceived usefulness. The term "Google" is often automatically cited and creates a feeling of complicity as though its use, in this context, has become part of a now widely shared private joke:

I: "Um, okay, and before your appointment with [Dr's name] can you remember whether you sought any sort of information or advice about your symptoms or?"
Pt:" Google." [interviewer laughs]
I: "Yeah."
Pt: Google. Yes, definitely Google. (Pat. 23)

Beyond highlighting that the internet constitutes an important step, patients also report being aware of a need to navigate the information it contains quite carefully. When asked why they prefaced their question to the GP with a disclaimer about having used the internet, a patient explained that

For peer review only - http://bmjopen.bmj.com/site/about/guidelines.xhtml

although they understood that unfiltered medical information can lead to unnecessary panic, it has become an almost instinctive step:

"*I repeat it, I know I shouldn't. Yeah 'cause as I said, you know, you tend to be a bit pessimistic and, uh, look at, um, whatever could be the worst thing linked to the, I don't know why, but for some reason, you know, you always look at, I think, the first thing is the, is that you look at it as a sort of first self-help, and, uh, there's been times where, maybe, I had something and I looked it up. And I thought, "Oh, phew, it's not that," as well, you know. Yeah. Erm, obviously cancer is the number one, um, and all sorts of things, but, um, yeah, I think, sometimes, internet can be useful."* (Pat. 28)

Linked to this is that although looking on the internet can be helpful there is a need to remain vigilant in terms of assessing the information, with a conclusion that consulting is the best solution to medical concerns.

"I repeat it, I know I shouldn't. […] I think, sometimes, internet can be useful. Erm, maybe, you know, if you have a little, um, problem, or you can look it up and you can have a better idea of what it is. Er, but again, you know, sometimes it can be on, on the other hand it could be quite misleading, because then you'll panic. So, it's two ways, you have to sort of know how to, probably how to use it and what to do with it. And always better to see a doctor, obviously." (Pat. 28)

Some people reported using the internet to solve their medical problems, and only when they failed to solve their problem 'resorted' to consulting a doctor.

"So, I did try and find out as much information as I could, but, erm, I wasn't very successful so, that's why. […] I resorted to going to the doctor. […] That's the only thing I did do, I just went on the internet, that's all." (Pat. 10)

Using the internet was described as serving a preparatory function in ensuring patients were equipped with information to facilitate their upcoming consultation. The internet was seen to present the offer of an advantage in terms of knowing the right questions to ask, enabling patients to navigate towards an optimal management or treatment outcome.

"You can ask the doctor more about what you've got because you've got more information about what you've got. I'm not saying that you distrust doctors or anything like that. But in this day and age with everybody under pressure, it's best to know what you're coming for. […] So it, you know, you wanna be able, you wanna have knowledge 'cause you wanna be able to not get fobbed off with anything less than getting [the issue] fixed. […]
"And in fact, they do say that people who are more eloquent with, with what they, how they can describe […] they get the better treatment. […] The doctors are highly intelligent, so if you go in there and […] have a good conversation, you're armed with information, you can ask the right questions and lead to the right treatment and try and get that treatment." (Pat. 6)

For peer review only - http://bmjopen.bmj.com/site/about/guidelines.xhtml

When asked what benefits they perceived from using the internet, some patients felt that the internet helped them to optimise limited time in the consultation room:

"You know, you've got limited time to get things across." (Pat. 3)

Finally, it is important to note that patients generally reported the need to reflect on their use of the internet. For example, in relation to when information available online would stop being useful and become instead a source of anxiety:

"But you really need to have, I think, a very steady mind [patient laughs] um, to use that. So, I kind of, um, rather than not looking on internet, I would say I look on internet, but keeping a sort of, um, protecting myself, you know, from going too far." (Pat. 28)

Use of the Internet as a reflection of the doctor-patient dynamic

In line with existing literature, [2, 9, 10] concern was expressed that it was not appropriate to mention use of the internet prior to consulting. This was associated with a perceived procedural etiquette when talking to their doctor.

I: "In the consultation I don't think you sort of referred to the fact that you'd gone on the internet, was there any reason why you didn't sort of bring that up or?"
Pt: "Um, I think I always think, doctors probably don't want to hear that, because you, you've gone to ask them."
I: "Mhm."
Pt:" Haven't you? So, um, I don't really want to say, "Well, I've looked it up on the internet what do you think?" (Pat. 23)

Patients talked of the need to monitor for the right slot to mention searching as well as a concern to ensure that mentioning use of the internet is not perceived as an explicit request or pressure for a particular GP action, such as prescribing.

"As I say, it's one of those things you sort of make known at the appropriate point if one arises. And not sort of, go in with a banner over your head saying, 'I've looked all this up on the internet. Now just give me a prescription brother'. Not the sensible way of doing it."" (Pat. 3) Some patients reported that their understanding of how interactions between doctors and patients worked meant they struggled to see how they could integrate what they learnt from the internet into the consultation.

"But on the whole, I don't think it's the sort of thing the GP wants to discuss. […] It doesn't fit my model, or what I perceive to be the GP's model, of how the conversation is supposed to go." (Pat. 11)

Interestingly the apparent sensitivity to discussing use of the internet for health was also evident in the patients' responses to the researcher, in which their internet use was presented as prudent,

For peer review only - http://bmjopen.bmj.com/site/about/guidelines.xhtml

rational, or "common sense", and in some cases patients emphasised legitimizing personal characteristics such as a professional background, for example healthcare or education, that worked to normalise their use of the internet

"So, I mean, because I was a teacher, just Googling and looking for information and sifting out information, something I did all the time, [...] as a (subject) teacher that you have to do that." (Pat. 6)

DISCUSSION:

Summary:

Patients reported use of the internet as an initial response to health symptoms and a way of reducing the pressure on the UK healthcare system. Their reports of soliciting medical expertise only after seeking information online, is consistent with previous research. [2] It is however notable that despite the internet being presented as a key way in which patients managed their health, caution was expressed by patients about both use and sharing internet use with the GP, and in some cases use of the internet was presented as a deviant action.

Strengths and limitations:

This analysis extends previous research about patients' use of, and views about, sharing information from the internet in primary care consultations. Only patients who agreed to have their consultation video recorded for the main study were invited to take part in the interview study. Of those, only patients for whom the internet was raised in either their pre-consultation survey, or their consultation were approached. However, this also serves to strengthen our findings in relation to patients' enduring concerns about discussing their internet use in general practice consultations.

Comparison with existing literature:

The findings are consistent with the existing literature regarding patients' reluctance to share details of their internet research despite internet use continuing to intensify over the years. [17, 18] Our findings are in line with Parsons' now classic sociological theory relating to medical interactions first outlined nearly 70 years ago. [19] Parsons pointed to the functional asymmetry of the medical encounter and how the medical consultation is itself embedded within the wider functionality of the institution of medicine in society. [19] While this may on the face of it appear undesirable and a barrier to open communication between the doctor and their patient, detailed analysis of medical interactions in a range of settings have illustrated that asymmetry is key to understanding the ways in which both medical professionals and their patients organize their interactions. [20] One way to engage with and position our findings is in relation to the idea discussed by Heritage [21] of access and rights to specific domains of medical knowledge and information in medical consultations. The avoidance by patients of broaching their use of the internet explicitly or directly with their healthcare providers can be seen as in keeping with attempts by patients to maintain the shared understandings in relation to how medical consultation operate and not be seen to encroach, uninvited, on the doctor's 'domain' of medical knowledge.

Implications for research and/or practice:

For peer review only - http://bmjopen.bmj.com/site/about/guidelines.xhtml

These observations highlight a difficulty in balancing the perceived responsibility of being a patient in the internet age on the one hand with the presentation of a feeling of needing to conform to a prior model in which 'medical knowledge' is only accessible via the doctor. There is an opportunity for GPs to take the initiative when patients present their problem at the beginning of the consultation to ask what they think might be wrong and if they have looked the problem up.

How this fits in:

Previous research on this topic showed that there was a reluctance on the part of the patients regarding sharing information found on the internet related to health concerns. Our findings support this claim and show that despite an increased democratisation and use of the internet in recent years since the publication of previous studies on this topic, this reluctance is still present. As internet use for health concerns by the general population is increasingly becoming common practice, it is relevant to explore the difficulty of the patient experience in the internet age. This study thus offers an insight into how patients might be approaching this subject, especially given the extent to which the current pandemic is and will continue to encourage a greater adoption of, reliance on, technology and the internet.

ACKNOWLEDGMENTS The authors would like to thank the participants of this study as well as the HaRI project research team for their support and collaboration. We would particularly like to acknowledge the work of Laura Hall who was one of the researchers on the study.

AUTHOR CONTRIBUTIONS: Study conception and design: all authors. Data collection: LH, MS. Manuscript Drafts: AC, FS. Comments on manuscript and agreement of drafts: all authors.

FUNDING: The Harnessing Resources from the Internet (HaRI) project was funded by the National Institute for Health Research School of Primary Care Research. The views expressed are those of the authors and not necessarily those of the NIHR, the NHS or the Department of Health. NHS costs are covered via the Local Clinical Research Network. The present study has received funding from the Laidlaw Foundation.

COMPETING INTERESTS: None declared.

ETHICS APPROVAL: The HaRI study received ethics approval from the Research Ethics Committee, University College London. All participants provided written consent before taking part in the study.

PROVENANCE AND PEER REVIEW Not commissioned; externally peer reviewed.

DATA SHARING STATEMENT Some data are available for reuse on application following appropriate ethics and data governance requirements.

PATIENT AND PUBLIC INVOLVEMENT

Two study participants and public involvement representatives advised the research group from the inception of the study. They were also members of the steering committee and advised throughout the study.

For peer review only - http://bmjopen.bmj.com/site/about/guidelines.xhtml

REFERENCES:

1. Oxford English Dictionary [Internet]. Oxford University Press; c2020. Internet; [cited 17 August 2019]; [about 1 screen]. Available from: https://www.oed.com/viewdictionaryentry/Entry/248411

2. Tan S, Goonawardene N. Internet Health Information Seeking and the Patient-Physician Relationship: A Systematic Review. *JMIR*. 2017;19(1):e9.

3. Prescott, C. Internet access – households and individuals, Great Britain - Office for National Statistics [Internet]. Ons.gov.uk. 2020 [cited 5 September 2018]. Available from: https://www.ons.gov.uk/peoplepopulationandcommunity/householdcharacteristics/homeinternetandsocialmediausage/bulletins/internetaccesshouseholdsandindividuals/2018

4. Empower the Person (formerly domain A) [Internet] NHS Digital. 2018. [Cited 5th September 2018]. Available from: https://digital.nhs.uk/about-nhs-digital/ourwork/transforming-health-and-carethrough-technology/empower-the-person-formerlydomain-a

5. Department of Health. Self-care – a real choice: self-care support — a practical option. London: HMSO, 2005.

6. '3 before GP': new RCGP mantra to help combat winter pressures in general practice [Internet]. Rcgp.org.uk. 2018 [cited 8 September 2018]. Available from: http://www.rcgp.org.uk/aboutus/news/2017/december/3-before-gp-new-rcgp-mantra-tohelp-combat-winter-pressures-ingeneral-practice.aspx

7. Dyer C. Doctors should not cherry pick what information to give patients, court rules BMJ 2015;350:h1414

8. "Dr Google enters 80% of my consultations", warns RCGP chair [Internet]. Available from:http://www.pulsetoday.co.uk/news/gp-topics/it/dr-google-enters-80-of-myconsultations-warnsrcgp-chair/20035400.article [Accessed 5 August 2019]

9. Bowes P, Stevenson F, Ahluwalia S and Murray E. 'I need her to be a doctor': patients' experiences of presenting health information from the internet in GP consultations. *Br J of Gen Pract*. 2012;732.

10. Ahluwalia S, Murray E, Stevenson F, Kerr C, Burns J. 'A heartbeat moment': qualitative study of GP views of patients bringing health information from the internet to a consultation. *Br J of Gen Pract*. 2010:60(571):88-94

For peer review only - http://bmjopen.bmj.com/site/about/guidelines.xhtml

11. C Bloor MJ, Hororbin GW. Conflict and conflict resolution in doctor/patient interactions. In: Ox C, Mead A, eds. A Sociology of Medical Practice. 1st ed. London: Collier Macmillan London; 1975. p.271-284.

12. Seguin M, Hall L, Atherton H, Barnes R, Leydon G, Murray E et al. Protocol paper for the 'Harnessing resources from the internet to maximise outcomes from GP consultations (HaRI)' study: a mixed qualitative methods study. *BMJ Open*. 2018;8(8):e024188.

13. Ritchie J, Lewis J. Qualitative research practice. Thousand Oaks, Calif.: SAGE; 2003.

14. Nowell L, Norris J, White D, Moules N. Thematic Analysis. *Int J Qual Methods.* 2017;16(1):1-13.

15. Department of Health & Social Care, National Information Board. Policy Paper: Personalised health and care 2020: a framework for action. Published 13 November 2014.

16. Braun V, Clarke V. Using thematic analysis in psychology. *Qual Res Psychol.* 2006;3(2):77-101.

17. Wang J, Ashvetiya T, Quaye E, Parakh X, Martin S S. Online Health Searches and Their Perceived Effects on Patients and Patient-Clinician Relationships: A Systematic Review. A*m J Med.* 2018;131(10).

18. Diviani N, Haukeland Fredriksen E, Meppelink C S, Mullan J, Rich W, Therkildsen Sudmann T. Where else would I look for it? A five-country qualitative study on purposes, strategies, and consequences of online health information seeking. *Journal of Public Health Research.* 2019;8(1): 33-39.

19. Parsons T. The social system. Psychology Press; 1991.

20. Pilnick A, Dingwall R. On the remarkable persistence of asymmetry in doctor/patient interaction: a critical review. *Soc Sci Med.* 2011;72(8):1374-1382. doi:10.1016/j.socscimed.2011.02.033

21. Heritage J (2012) Epistemic in conversation in Sidnell, J. & Stivers, T. (Eds.) The Handbook of Conversation Analysis. Blackwell-Wiley P 370-394.

For peer review only - http://bmjopen.bmj.com/site/about/guidelines.xhtml

SUPPLEMENTAL MATERIAL 1 – INTERVIEW TOPIC GUIDE

Interview Topic Guide: Patients

Topic Guide : Interviews with patients

Harnessing resources from the internet to maximise outcomes from GP consultations (HaRI): a qualitative study

1. Consultation

Could you start by describing what you can remember about the consultation we videorecorded.

2. Sources of information and advice

Before your appointment with [GP's name], did you seek information or advice about symptoms, conditions, treatments or health services?

INTERNET MENTIONED

Focusing on the information/advice you looked for on the internet, can you remember what information/advice you were looking for?

Did you refer to the information/advice you looked for on the internet during your consultation with [GP's name]?

-Yes

How did the doctor respond?

-No

Was there any reason why you did not refer to the information/advice you looked for on the internet during your consultation?

NO MENTION OF INTERNET

Did you consult the internet for information or advice about symptoms, conditions, treatments or health services before your appointment with [GP's name]?

-Yes

Did you refer to this during your consultation?

-Yes

For peer review only

How did the doctor respond?

-No

Was there any reason why you did not refer to the information/advice you looked for on the internet during your consultation?

…………………………………………………………………………………………..

-No

Have you looked for information or advice about symptoms, conditions, treatments or health services on the internet before your consultation with the doctor on some other occasion?

YES

Did you refer to this during your consultation?

-Yes

How did the doctor respond?

-No

Was there any reason why you did not refer to the information or advice you looked for on the internet during your consultation?

3. GP use of internet for diagnosis/treatment recommendation

Has the doctor ever looked something up on the internet during your consultation?

-Condition

-Treatment

YES

-Can you remember any particular occasions?

-What were your thoughts on the doctor doing that?

NO

-What do you think about doctors looking up information on the internet during consultations?

4. GP use of internet for explaining condition/treatment

Has the doctor ever used information from the internet to help him/her explain a condition or treatment?

-Talked through information on a webpage

For peer review only

-Images

**YES**
-Can you remember any particular occasions when your doctor has done this?
-What were your thoughts on the doctor doing that?

**NO**
What do you think about doctors using information from the internet to help explain a condition or treatment during consultations?

5. GP printing out information from the internet for patient to take away

Has the doctor ever printed out information from the internet about a health condition or treatment for you to take away and read?

**YES**
-Can you remember any particular occasions when your doctor has done this?
-What were your thoughts on the doctor doing that?

**NO**
What do you think about doctors printing out information from the internet for patients to take away and read?

6. GP recommendation

Has the doctor ever recommended a particular website during your consultation?

**YES**
-Can you remember any particular occasions when your doctor has done this?
-What were your thoughts on the doctor doing that?

7. GP warning

Has the doctor ever suggested that you don't look for information or advice on the internet?

**YES**
-What did you think about the doctor doing that?

For peer review only

NO

-What do you think about doctors suggesting to patients that they should not look for health information or advice on the internet?

8. Anything to add

Is there anything you would like to add about:

-The information patients and GPs use in and outside of consultations to manage health problems

-GP consultations more generally

9. Reflections on taking part in the research

How did you find participating in the study?

For peer review only

SUPPLEMENTAL MATERIAL 2 – CONSENT FORM

Title of Project: **Harnessing resources to maximise outcomes from GP consultations (HaRI): a qualitative study**

Please initial all boxes

1. I confirm that I have read and understand the information sheet provided dated 15th July 2016 (v 1.2) for the above study. I have had the opportunity to consider the information, ask questions and have had these answered satisfactorily. ☐

2. I understand that my participation is voluntary and that I am free to withdraw at any time up until the end of data collection at this site without giving any reason. ☐

3. I understand that my interview will be audio-recorded. ☐

4. I understand that the interview will be transcribed by a professional transcribing company with details anonymised to protect my identity. ☐

5. I agree to the use of anonoymised verbatim quotation for the purposes of education and teaching (for example publication, conference presentations). ☐

CONSENT TO POINTS 6 AND 7 IS OPTIONAL AND NOT REQUIRED FOR STUDY PARTICIPATION

6. I consent for the transcript of the interview to be placed into an archive at UCL to allow data to be used, subject to an appropriate protocol and ethical approval, by other researchers. I understand this carries a potential risk that I might be identified. ☐

7. I consent for the audio tape to be placed into an archive at UCL to allow data to be used, subject to an appropriate protocol and ethical approval, by other researchers. I understand this carries a potential risk that I might be identified. ☐

____________________	____________________	____________________
Name of Participant	Date (DD/MM/YY)	Signature
____________________	____________________	____________________
Name of person taking consent	Date (DD/MM/YY)	Signature

For peer review only - http://bmjopen.bmj.com/site/about/guidelines.xhtml

COREQ (COnsolidated criteria for REporting Qualitative research) Checklist

A checklist of items that should be included in reports of qualitative research. You must report the page number in your manuscript where you consider each of the items listed in this checklist. If you have not included this information, either revise your manuscript accordingly before submitting or note N/A.

Topic	Item No.	Guide Questions/Description	Reported on Page No.
**Domain 1: Research team and reflexivity**			
Personal characteristics			
Interviewer/facilitator	1	Which author/s conducted the interview or focus group?	
Credentials	2	What were the researcher's credentials? E.g. PhD, MD	
Occupation	3	What was their occupation at the time of the study?	
Gender	4	Was the researcher male or female?	
Experience and training	5	What experience or training did the researcher have?	
Relationship with participants			
Relationship established	6	Was a relationship established prior to study commencement?	
Participant knowledge of the interviewer	7	What did the participants know about the researcher? e.g. personal goals, reasons for doing the research	
Interviewer characteristics	8	What characteristics were reported about the inter viewer/facilitator? e.g. Bias, assumptions, reasons and interests in the research topic	
**Domain 2: Study design**			
Theoretical framework			
Methodological orientation and Theory	9	What methodological orientation was stated to underpin the study? e.g. grounded theory, discourse analysis, ethnography, phenomenology, content analysis	
Participant selection			
Sampling	10	How were participants selected? e.g. purposive, convenience, consecutive, snowball	
Method of approach	11	How were participants approached? e.g. face-to-face, telephone, mail, email	
Sample size	12	How many participants were in the study?	
Non-participation	13	How many people refused to participate or dropped out? Reasons?	
Setting			
Setting of data collection	14	Where was the data collected? e.g. home, clinic, workplace	
Presence of non-participants	15	Was anyone else present besides the participants and researchers?	
Description of sample	16	What are the important characteristics of the sample? e.g. demographic data, date	
Data collection			
Interview guide	17	Were questions, prompts, guides provided by the authors? Was it pilot tested?	
Repeat interviews	18	Were repeat inter views carried out? If yes, how many?	
Audio/visual recording	19	Did the research use audio or visual recording to collect the data?	
Field notes	20	Were field notes made during and/or after the inter view or focus group?	
Duration	21	What was the duration of the inter views or focus group?	
Data saturation	22	Was data saturation discussed?	
Transcripts returned	23	Were transcripts returned to participants for comment and/or	

For peer review only

Topic	Item No.	Guide Questions/Description	Reported on Page No.
		correction?	
**Domain 3: analysis and findings**			
Data analysis			
Number of data coders	24	How many data coders coded the data?	
Description of the coding tree	25	Did authors provide a description of the coding tree?	
Derivation of themes	26	Were themes identified in advance or derived from the data?	
Software	27	What software, if applicable, was used to manage the data?	
Participant checking	28	Did participants provide feedback on the findings?	
Reporting			
Quotations presented	29	Were participant quotations presented to illustrate the themes/findings? Was each quotation identified? e.g. participant number	
Data and findings consistent	30	Was there consistency between the data presented and the findings?	
Clarity of major themes	31	Were major themes clearly presented in the findings?	
Clarity of minor themes	32	Is there a description of diverse cases or discussion of minor themes?	

Developed from: Tong A, Sainsbury P, Craig J. Consolidated criteria for reporting qualitative research (COREQ): a 32-item checklist for interviews and focus groups. *International Journal for Quality in Health Care*. 2007. Volume 19, Number 6: pp. 349 – 357

Once you have completed this checklist, please save a copy and upload it as part of your submission. DO NOT include this checklist as part of the main manuscript document. It must be uploaded as a separate file.

For peer review only

Standards for Reporting Qualitative Research (SRQR)*

http://www.equator-network.org/reporting-guidelines/srqr/

Page/line no(s).

Title and abstract

**Title** - Concise description of the nature and topic of the study Identifying the study as qualitative or indicating the approach (e.g., ethnography, grounded theory) or data collection methods (e.g., interview, focus group) is recommended	p.1 / l.2-3
**Abstract** - Summary of key elements of the study using the abstract format of the intended publication; typically includes background, purpose, methods, results, and conclusions	p.3/l.1-24

Introduction

**Problem formulation** - Description and significance of the problem/phenomenon studied; review of relevant theory and empirical work; problem statement	p.3/l.25-p.4/l.12
**Purpose or research question** - Purpose of the study and specific objectives or questions	p.4/l.14

Methods

**Qualitative approach and research paradigm** - Qualitative approach (e.g., ethnography, grounded theory, case study, phenomenology, narrative research) and guiding theory if appropriate; identifying the research paradigm (e.g., postpositivist, constructivist/ interpretivist) is also recommended; rationale**	p.5/l.13-21
**Researcher characteristics and reflexivity** - Researchers' characteristics that may influence the research, including personal attributes, qualifications/experience, relationship with participants, assumptions, and/or presuppositions; potential or actual interaction between researchers' characteristics and the research questions, approach, methods, results, and/or transferability	p.5/l.15-18
**Context** - Setting/site and salient contextual factors; rationale**	p.4/l.26-28
**Sampling strategy** - How and why research participants, documents, or events were selected; criteria for deciding when no further sampling was necessary (e.g., sampling saturation); rationale**	p.4/l.36- p.5/l.2
**Ethical issues pertaining to human subjects** - Documentation of approval by an appropriate ethics review board and participant consent, or explanation for lack thereof; other confidentiality and data security issues	p.4/l.31-33
**Data collection methods** - Types of data collected; details of data collection procedures including (as appropriate) start and stop dates of data collection and analysis, iterative process, triangulation of sources/methods, and modification of procedures in response to evolving study findings; rationale**	

For peer review only - http://bmjopen.bmj.com/site/about/guidelines.xhtml

**Data collection instruments and technologies** - Description of instruments (e.g., interview guides, questionnaires) and devices (e.g., audio recorders) used for data collection; if/how the instrument(s) changed over the course of the study	p.5/l.5-10.
**Units of study** - Number and relevant characteristics of participants, documents, or events included in the study; level of participation (could be reported in results)	p.4/l.31
**Data processing** - Methods for processing data prior to and during analysis, including transcription, data entry, data management and security, verification of data integrity, data coding, and anonymization/de-identification of excerpts	p.5/l.8
**Data analysis** - Process by which inferences, themes, etc., were identified and developed, including the researchers involved in data analysis; usually references a specific paradigm or approach; rationale**	p.5/l.13
**Techniques to enhance trustworthiness** - Techniques to enhance trustworthiness and credibility of data analysis (e.g., member checking, audit trail, triangulation); rationale**	p.5/l.19

Results/findings

**Synthesis and interpretation** - Main findings (e.g., interpretations, inferences, and themes); might include development of a theory or model, or integration with prior research or theory	p.6/l.2-8<br>p.8/l.14-17
**Links to empirical data** - Evidence (e.g., quotes, field notes, text excerpts, photographs) to substantiate analytic findings	p.6/l.10-p.8/l.12<br>p.8/l.19-p.9/l.7

Discussion

**Integration with prior work, implications, transferability, and contribution(s) to the field -** Short summary of main findings; explanation of how findings and conclusions connect to, support, elaborate on, or challenge conclusions of earlier scholarship; discussion of scope of application/generalizability; identification of unique contribution(s) to scholarship in a discipline or field	p.9/l.11-19<br>p.9/l.25-39<br>p.10/l.1-5
**Limitations** - Trustworthiness and limitations of findings	p.9/l.19-23

Other

**Conflicts of interest** - Potential sources of influence or perceived influence on study conduct and conclusions; how these were managed	p.10/l.40
**Funding** - Sources of funding and other support; role of funders in data collection, interpretation, and reporting	p.10/l.34-38

*The authors created the SRQR by searching the literature to identify guidelines, reporting standards, and critical appraisal criteria for qualitative research; reviewing the reference lists of retrieved sources; and contacting experts to gain feedback. The SRQR aims to improve the transparency of all aspects of qualitative research by providing clear standards for reporting qualitative research.

For peer review only

**The rationale should briefly discuss the justification for choosing that theory, approach, method, or technique rather than other options available, the assumptions and limitations implicit in those choices, and how those choices influence study conclusions and transferability. As appropriate, the rationale for several items might be discussed together.

__Reference:__
O'Brien BC, Harris IB, Beckman TJ, Reed DA, Cook DA. **Standards for reporting qualitative research: a synthesis of recommendations.** *Academic Medicine*, Vol. 89, No. 9 / Sept 2014 DOI: 10.1097/ACM.0000000000000388

For peer review only

For peer review only - http://bmjopen.bmj.com/site/about/guidelines.xhtml

BMJ Open

Qualitative Study: Patients' Enduring Concerns about Discussing Internet Use in General Practice Consultations

Journal:	*BMJ Open*
Manuscript ID	bmjopen-2020-047508.R1
Article Type:	Original research
Date Submitted by the Author:	24-Mar-2021

Complete List of Authors:	Cuteanu, Anita; University College London Research Department of Primary Care and Population Health,<br>Seguin, Maureen ; London School of Hygiene and Tropical Medicine<br>Ziebland, Sue; University of Oxford, NDPCHS<br>Pope, Catherine; University of Oxford, Nuffield Department of Primary Care Health Sciences<br>Leydon, Geraldine; University of Southampton, Faculty of Medicine<br>Barnes, Rebecca; Bristol University, Centre for Academic Primary Care, School for Social and Community Medicine<br>Murray, Elizabeth; University College London Research Department of Primary Care and Population Health<br>Atherton, Helen; University of Warwick,<br>Stevenson, Fiona; University College London Research Department of Primary Care and Population Health

**Primary Subject Heading**:	Public health
Secondary Subject Heading:	Qualitative research
Keywords:	PRIMARY CARE, QUALITATIVE RESEARCH, World Wide Web technology < BIOTECHNOLOGY & BIOINFORMATICS

SCHOLARONE™ Manuscripts

I, the Submitting Author has the right to grant and does grant on behalf of all authors of the Work (as defined in the below author licence), an exclusive licence and/or a non-exclusive licence for contributions from authors who are: i) UK Crown employees; ii) where BMJ has agreed a CC-BY licence shall apply, and/or iii) in accordance with the terms applicable for US Federal Government officers or employees acting as part of their official duties; on a worldwide, perpetual, irrevocable, royalty-free basis to BMJ Publishing Group Ltd ("BMJ") its licensees and where the relevant Journal is co-owned by BMJ to the co-owners of the Journal, to publish the Work in this journal and any other BMJ products and to exploit all rights, as set out in our licence.

The Submitting Author accepts and understands that any supply made under these terms is made by BMJ to the Submitting Author unless you are acting as an employee on behalf of your employer or a postgraduate student of an affiliated institution which is paying any applicable article publishing charge ("APC") for Open Access articles. Where the Submitting Author wishes to make the Work available on an Open Access basis (and intends to pay the relevant APC), the terms of reuse of such Open Access shall be governed by a Creative Commons licence – details of these licences and which Creative Commons licence will apply to this Work are set out in our licence referred to above.

Other than as permitted in any relevant BMJ Author's Self Archiving Policies, I confirm this Work has not been accepted for publication elsewhere, is not being considered for publication elsewhere and does not duplicate material already published. I confirm all authors consent to publication of this Work and authorise the granting of this licence.

For peer review only

Main Document

Title: Qualitative Study: Patients' Enduring Concerns about Discussing Internet Use in General Practice Consultations

Authors: Cuteanu Anita, Seguin Maureen, Ziebland Sue, Pope Catherine, Leydon Geraldine, Barnes Rebecca, Murray Elizabeth, Atherton Helen, Stevenson Fiona.

1- Ms Anita Cuteanu
(Corresponding Author)
a.cuteanu@ucl.ac.uk
1. Laidlaw Fellow,
University College London Research Department of Primary Care and Population Health
Gower St, Bloomsbury
London, London, UK NW3 2PF

2- Dr Maureen Seguin, BA, MA, PhD
Maureen.Seguin@lshtm.ac.uk
1. Research Fellow,
London School of Hygiene & Tropical Medicine
London, London, UK WC1E 7HT

3- Professor Sue Ziebland, BA, MSc, PhD
sue.ziebland@phc.ox.ac.uk
1. Professor of Medical Sociology,
University of Oxford Nuffield Department of Primary Care Health Sciences
Oxford, Oxfordshire, UK OX3 7LF

4- Professor Catherine Pope, BA (Hons), PhD
catherine.pope@phc.ox.ac.uk
1. Professor of Medical Sociology
University of Oxford, Nuffield Department of Primary Care Health Sciences
Oxford, Oxfordshire, UK OX1 2JD

5- Professor Geraldine Leydon, BA, MA, PhD, Dip. PH
G.M.Leydon@soton.ac.uk
1. University of Southampton
Southampton, Hampshire, UK SO17 1BJ

6- Dr Rebecca Kate Barnes, BSc(Hons), PGDip., PhD
Rebecca.Barnes@bristol.ac.uk
0000-0001-8844-7496
1. Senior Research Fellow
University of Bristol, Bristol Medical School, Population Health Sciences
Canynge Hall

For peer review only

39 Whatley Rd
Bristol, UK BS8 2PS

7- Professor Elizabeth Murray, FRCGP FRCP(Edin) PhD
elizabeth.murray@ucl.ac.uk
1. Professor of e-Health and Primary Care
University College London Research Department of Primary Care and Population Health
London, London, UK NW3 2PF

8- Dr Helen Atherton, BSc, MSc, PhD
h.atherton@warwick.ac.uk
0000-0002-7072-1925
1. Associate Professor
University of Warwick, Warwick Medical School
Gibbet Hill Road
Coventry, UK CV4 7AL

9- Professor Fiona Stevenson, MA (Hons), PgDip., PhD
f.stevenson@ucl.ac.uk
1. Professor of Medical Sociology,
University College London Research Department of Primary Care and Population Health
Gower St, Bloomsbury
London, London, UK NW3 2PF

Word count: 2965 words.

For peer review only

ABSTRACT:

Objectives: To examine patients' accounts of their use of the internet before seeing a general practitioner (GP) using thematic analysis of semi-structured interviews.

Design: Qualitative semi-structured interview study with transcripts analysed thematically.

Setting: Primary care patients consulting with 10 GPs working at 7 GP practices of varying sizes and at a range of locations around London and the Southeast of England.

Participants: 28 adult patients: 16 female and 12 male ranging in age from 18 to 75 from a range of self-defined ethnic backgrounds. Participants were selected based on instances when the patients reported having used the internet before the consultation, when patients referred to the internet in the consultation or when the physician used the internet or made reference to it during the consultation.

Results: Patients report that they can find health information online which they believe is reliable and helpful for both themselves and their GP. However, they report uncertainty about how to share internet-based findings and reluctance to disclose their efforts at researching health issues online for fear of appearing disrespectful or interfering with the flow of the consultation.

Conclusions: Despite the democratisation of access to information about health due via the internet patients continue to experience their use of the internet for health information as a sensitive and potentially problematic topic. The onus may well be on GPs to raise the likelihood (without judgement) that patients will have looked things up before consulting and invite them to talk about what they found.

STRENGTHS AND LIMITATIONS OF THIS STUDY

- This study serves to strengthen findings in relation to patients' enduring concerns about discussing their internet use in general practice consultations.
- Only patients who agreed to have their consultation video recorded for the main study ('Harnessing resources from the internet to maximise outcomes from GP consultations' (HaRI)) were invited to take part in the interview study.
- Of those, only patients for whom the internet was raised in either their pre-consultation survey, or their consultation, were approached.

INTRODUCTION:

The internet, the "global computer network" [1] connecting and providing us with unlimited information at our fingertips, has become increasingly present in everyday life, with daily use having more than doubled in the last twelve years in the U.K. [2, 3] It is not surprising that its use for health-related information has followed the same trend, [2] especially as British health authorities and successive governments have increasingly encouraged the population to become more proactive and independent regarding their own healthcare and health management. [3-6] In summary, the internet has become an important source of a wide range of medical information

For peer review only - http://bmjopen.bmj.com/site/about/guidelines.xhtml

which is easily accessible by those without medical training. [2, 7] Indeed, Professor Helen Stokes-Lampard, former Chair of the Royal College of General Practitioners highlighted the increasing pervasiveness of the internet in consultations, dubbing its presence as that of "Dr. Google". [8] However, while patients are being encouraged to take on more responsibility and play a greater role in their own health care, both GPs and patients are concerned about how discussions of internet-based health information affect relationships in the consultation. [9,10] Patients report a desire to maximise the available time in consultations by preparing in advance and informing themselves about their problems and possible solutions. [9] Nevertheless, they remain mindful of not wanting to appear to challenge their GP's expertise. [9] Conversely, GPs express anxiety about the potential for their status and expertise to be called into question. [10] In some cases, patients may even find themselves trapped, to at least some degree, in what Bloor and Hororbin described as a "double-bind" which expects them to be sufficiently informed, but still defer to their GP's medical expertise and feign ignorance in relation to their medical problem. [11]

This paper offers an in-depth examination of patients' accounts concerning their use of the internet before, during and following GP consultations. Given the aforementioned persistent unease surrounding the use of the internet in the consultation room, we reflect on the ways in which patients discuss and account for their use of the internet with regard to general practice consultations.

METHODS

Design

Semi structured qualitative interviews were conducted with 28 patients as part of the Harnessing Resources from the Internet (HaRI) study. [12] The complete data set comprises 281 video-recorded general practice (GP) consultations, with pre-consultation questionnaires completed by all patients, interviews with all 10 participating doctors and 28 selected patients. The 10 GPs came from seven practices across London and the southeast of England. Practices varied in terms of size, population density, level of deprivation and whether they were a training practice. The interviews sought to capture patients' accounts of their use of the internet prior to their video recorded consultation and reflect on how and why they might, or might not, share prior use of the internet in consultations with their GP.

Data

We focus on the 28 patients who were interviewed after the consultation. Data were collected between September 2017 and May 2018. Ethical Approval was obtained from the UK NHS Research Ethics Committee, and governance approval obtained from the Health Research Authority.

Data collection

For the initial data collection, up to five patients who had previously indicated they would be prepared to be interviewed, were purposively selected (on the basis of survey and consultation data) from each practice and approached. Patients were selected to reflect (i) instances in which patients reported searching the internet before their GP consultation and this was raised in the consultation, (ii) instances in which patients reported searching the internet before their GP

For peer review only - http://bmjopen.bmj.com/site/about/guidelines.xhtml

consultation and this was NOT raised in the consultation, (iii) instances in which the patient raised the topic of the internet in the consultation and this was not reported in the pre-consultation questionnaire and (iv) instances in which the GP used the internet or raised it as a topic during the consultation.

We sought to provide maximum variation in terms of socio-demographic characteristics. All participants provided signed informed consent which included permission to publish anonymised extracts for the purpose of education and teaching (See Supplemental Material 1). To minimise any risk of coercion, it was made clear that the decision to participate or to decline would not affect clinical care and that it was possible to withdraw consent up to the time all data were collected at their site. We note that in one of the practices, practice number five, no patients agreed to be interviewed. GPs were not told which patients had been approached for a subsequent interview.

Semi-structured interviews were conducted by one of two experienced female, non-clinical, qualitative researchers. They took place either in patients' homes or, if preferred, in a room provided by their GP practice. Interviews varied in length from 25 to 90 minutes and were audio recorded. All were based on an agreed topic guide (See Supplemental Material 2), which focused on accounts of what patients did prior to consulting, with prompts relating to the internet if it was not raised. Patients were also asked what happened in the consultation. The interviews were transcribed verbatim and anonymised by a professional transcription service with the transcripts checked for accuracy by the team.

Data analysis

Data were analysed using thematic analysis [13, 14, 15]. Data analysis began with the process of familiarisation, noting down general impressions and providing a content summary for each interview. Initial codes and impressions were carefully sorted and collated into overarching themes. [16] Employing, as Nowell et al. [16] put it, "an iterative and reflective process that develops over time and involves a constant moving back and forward between phases" [16, p.4] A series of coherent themes were developed, reviewed and defined over a period of several weeks. A data clinic session was held with seven experienced qualitative researchers to help further refine and strengthen the existing themes. Additional discussions and brainstorming sessions with the project leader (FS) helped structure the themes and develop the narrative.

FINDINGS

Participant characteristics:

Interviewees comprised 16 female and 12 male patients ranging in age from 18 to 75 years of age (see Table 1).

Table 1: Characteristics of patients interviewed

Gender	Age	Condition
M	56 - 65	Foot problem
F	56 - 65	Urinary tract infection
M	56 - 65	Suspected inner-ear benign tumour.
F	56 - 65	Blood in stools

For peer review only - http://bmjopen.bmj.com/site/about/guidelines.xhtml

F	19 - 25	Throat feel bruised
F	56 - 65	Blood test result
M	46 - 55	Knee pain.
F	46 - 55	Shoulder pain
M	56 - 65	Suspected hernia.
F	66 - 75	Requested knee replacement.
M	66 - 75	Hole where hernia operation scar is.
M	66 - 75	PSA test result
F	36 - 55	Thyroid problem
F	66 - 75	Hissing sound in one ear
F	26 - 35	Pain in ear.
F	26 - 35	Complications from deviated septum surgery
M	0 -18	Baby has oozing belly button
F	66 - 75	Respiratory infection
F	66 - 75	UTI and Heart issues
F	56 - 65	Pain behind knee.
M	0 -18	Three-year-old boy has spot on neck.
M	46 - 55	Discussion of patient's recovery from throat cancer.
F	56 - 65	Review of patient's overactive thyroid.
M	46 - 55	Lethargy, pain in chest.
M	56 - 65	Check up for diabetes and.
M	66 - 75	Review of pain in head.
F	46 - 55	Lower back pain, numbness in tip of middle finger.
F	46 - 55	Mark on chest

Internet Research – A Complicated but Necessary First Step for Patients?
Although the literature suggests that patients are worried about being perceived negatively, in this study most patients presented consulting the internet as an important and necessary step prior to seeing their GP. [2] Researching symptoms or concerns on the internet via the well-known web search engine "Google" has become a reflex for many. This is a testament to its perceived usefulness. The term "Google" is often automatically cited and creates a feeling of complicity as though its use, in this context, has become part of a now widely shared private joke:

For peer review only - http://bmjopen.bmj.com/site/about/guidelines.xhtml

I: "Um, okay, and before your appointment with [Dr's name] can you remember whether you sought any sort of information or advice about your symptoms or?"
Pt:" Google." [interviewer laughs]
I: "Yeah."
Pt: Google. Yes, definitely Google. (Pat. 23)

Beyond highlighting that the internet constitutes an important step, patients also report being aware of a need to navigate the information it contains quite carefully. When asked why they prefaced their question to the GP with a disclaimer about having used the internet, a patient explained that although they understood that unfiltered medical information can lead to unnecessary panic, it has become an almost instinctive step:

"*I repeat it, I know I shouldn't. Yeah 'cause as I said, you know, you tend to be a bit pessimistic and, uh, look at, um, whatever could be the worst thing linked to the, I don't know why, but for some reason, you know, you always look at, I think, the first thing is the, is that you look at it as a sort of first self-help, and, uh, there's been times where, maybe, I had something and I looked it up. And I thought, "Oh, phew, it's not that," as well, you know. Yeah. Erm, obviously cancer is the number one, um, and all sorts of things, but, um, yeah, I think, sometimes, internet can be useful."* (Pat. 28)

Linked to this is that although looking on the internet can be helpful there is a need to remain vigilant in terms of assessing the information, with a conclusion that consulting is the best solution to medical concerns.

"I think, sometimes, internet can be useful. Erm, maybe, you know, if you have a little, um, problem, or you can look it up and you can have a better idea of what it is. Er, but again, you know, sometimes it can be on, on the other hand it could be quite misleading, because then you'll panic. So, it's two ways, you have to sort of know how to, probably how to use it and what to do with it. And always better to see a doctor, obviously." (Pat. 28)

Some people reported using the internet to solve their medical problems, and only when they failed to solve their problem 'resorted' to consulting a doctor.

"So, I did try and find out as much information as I could, but, erm, I wasn't very successful so, that's why. […] I resorted to going to the doctor. […] That's the only thing I did do, I just went on the internet, that's all." (Pat. 10)

Using the internet was described as serving a preparatory function in ensuring patients were equipped with information to facilitate their upcoming consultation. The internet was seen to present the offer of an advantage in terms of knowing the right questions to ask, enabling patients to navigate towards an optimal management or treatment outcome.

For peer review only - http://bmjopen.bmj.com/site/about/guidelines.xhtml

"You can ask the doctor more about what you've got because you've got more information about what you've got. I'm not saying that you distrust doctors or anything like that. But in this day and age with everybody under pressure, it's best to know what you're coming for. […] So it, you know, you wanna be able, you wanna have knowledge 'cause you wanna be able to not get fobbed off with anything less than getting [the issue] fixed. […]
"And in fact, they do say that people who are more eloquent with, with what they, how they can describe […] they get the better treatment. […] The doctors are highly intelligent, so if you go in there and […] have a good conversation, you're armed with information, you can ask the right questions and lead to the right treatment and try and get that treatment." (Pat. 6)

When asked what benefits they perceived from using the internet, some patients felt that the internet helped them to optimise limited time in the consultation room:

"You know, you've got limited time to get things across." (Pat. 3)

Finally, it is important to note that patients generally reported the need to reflect on their use of the internet. For example, in relation to when information available online would stop being useful and become instead a source of anxiety:

"But you really need to have, I think, a very steady mind [patient laughs] um, to use that. So, I kind of, um, rather than not looking on internet, I would say I look on internet, but keeping a sort of, um, protecting myself, you know, from going too far." (Pat. 28)

Use of the Internet as a reflection of the doctor-patient dynamic
In line with existing literature, [2, 9, 10] concern was expressed that it was not appropriate to mention use of the internet prior to consulting. This was associated with a perceived procedural etiquette when talking to their doctor.

I: "In the consultation I don't think you sort of referred to the fact that you'd gone on the internet, was there any reason why you didn't sort of bring that up or?"
Pt: "Um, I think I always think, doctors probably don't want to hear that, because you, you've gone to ask them."
I: "Mhm."
Pt:" Haven't you? So, um, I don't really want to say, "Well, I've looked it up on the internet what do you think?" (Pat. 23)

Patients talked of the need to monitor for the right slot to mention searching as well as a concern to ensure that mentioning use of the internet is not perceived as an explicit request or pressure for a particular GP action, such as prescribing.

"As I say, it's one of those things you sort of make known at the appropriate point if one arises. And not sort of, go in with a banner over your head saying, 'I've looked all this up on the internet. Now just give me a prescription brother'. Not the sensible way of doing it."" (Pat. 3)

For peer review only - http://bmjopen.bmj.com/site/about/guidelines.xhtml

Some patients reported that their understanding of how interactions between doctors and patients worked meant they struggled to see how they could integrate what they learnt from the internet into the consultation.

"But on the whole, I don't think it's the sort of thing the GP wants to discuss. […] It doesn't fit my model, or what I perceive to be the GP's model, of how the conversation is supposed to go." (Pat. 11)

Interestingly the apparent sensitivity to discussing use of the internet for health was also evident in the patients' responses to the researcher, in which their internet use was presented as prudent, rational, or "common sense", and in some cases patients emphasised legitimizing personal characteristics such as a professional background, for example healthcare or education, that worked to normalise their use of the internet

"So, I mean, because I was a teacher, just Googling and looking for information and sifting out information, something I did all the time, [...] as a (subject) teacher that you have to do that." (Pat. 6)

DISCUSSION:

Summary:

Patients reported use of the internet as an initial response to health symptoms and a way of reducing the pressure on the UK healthcare system. Their reports of soliciting medical expertise only after seeking information online is consistent with previous research. [2] It is however notable that despite the internet being presented as a key way in which patients managed their health, caution was expressed by patients about both use and sharing internet use with the GP, and in some cases use of the internet was presented as a deviant action.

Strengths and limitations:

This analysis extends previous research about patients' use of, and views about, sharing information from the internet in primary care consultations. Only patients who agreed to have their consultation video recorded for the main study and indicated they were happy to be contacted for an interview were invited to take part in the interview study. Of those, only patients for whom the internet was raised in either their pre-consultation survey, or their consultation, were approached. This means our sample focused on those people for whom the internet had played a part in a recent consultation grounding data collection in an actual event as opposed to hypothetical ideas.

Comparison with existing literature:

The findings are consistent with the existing literature regarding patients' reluctance to share details of their internet research despite internet use continuing to intensify over the years. [17, 18] Our findings are in line with Parsons' now classic sociological theory relating to medical interactions first outlined nearly 70 years ago. [19] Parsons pointed to the functional asymmetry of the medical encounter and how the medical consultation is itself embedded within the wider functionality of the institution of medicine in society. [19] While this may on the face of it appear

For peer review only - http://bmjopen.bmj.com/site/about/guidelines.xhtml

undesirable and a barrier to open communication between the doctor and their patient, detailed analysis of medical interactions in a range of settings have illustrated that asymmetry is key to understanding the ways in which both medical professionals and their patients organize their interactions. [20] One way to engage with and position our findings is in relation to the idea discussed by Heritage [21] of access and rights to specific domains of medical knowledge and information in medical consultations. The avoidance by patients of broaching their use of the internet explicitly or directly with their healthcare providers can be seen as in keeping with attempts by patients to maintain the shared understandings in relation to how medical consultation operate and not be seen to encroach, uninvited, on the doctor's 'domain' of medical knowledge.

Implications for research and/or practice:

These observations highlight a difficulty in balancing the perceived responsibility of being a patient in the internet age on the one hand with the presentation of a feeling of needing to conform to a prior model in which 'medical knowledge' is only accessible via the doctor. There is an opportunity for GPs to take the initiative when patients present their problem at the beginning of the consultation to ask what they think might be wrong and if they have looked the problem up.

CONCLUSION

Previous research on this topic showed that there was a reluctance on the part of patients regarding sharing information found on the internet. Our findings support this claim and show that despite an ever increasing democratisation of access to information and growth in use of the internet across the population this reluctance is still present. This study offers insights into how patients approaching use of the internet for health concerns. Such understandings are crucial given the current pandemic is and will continue to encourage a greater adoption of, reliance on, technology and the internet in particular.

ACKNOWLEDGMENTS The authors would like to thank the participants of this study as well as the HaRI project research team for their support and collaboration. We would particularly like to acknowledge the work of Laura Hall who was one of the researchers on the study.

AUTHOR CONTRIBUTIONS: FS, GL, RB, SZ, CP, EM, HA conceived of the study, designed it and securing funding. MS collected the data. Analysis of the data and initial drafts were led by AC and FA and the manuscript was reviewed critically by GL, RB, SZ, CP, EM, HA, MS. All authors have edited and approved the manuscript for submission.

FUNDING: The Harnessing Resources from the Internet (HaRI) project was funded by the National Institute for Health Research School for Primary Care Research (Award/Grant Number 284). The views expressed are those of the authors and not necessarily those of the NIHR, the NHS or the Department of Health. NHS costs are covered via the Local Clinical Research Network. The present study has received funding from the Laidlaw Foundation.

COMPETING INTERESTS: None declared.

For peer review only - http://bmjopen.bmj.com/site/about/guidelines.xhtml

ETHICS APPROVAL: The HaRI study received ethics approval from the UK NHS Research Ethics Committee and governance approval obtained from the Health Research Authority. All participants provided written consent before taking part in the study.

PROVENANCE AND PEER REVIEW Not commissioned; externally peer reviewed.

DATA SHARING STATEMENT Some data are available for reuse on application following appropriate ethics and data governance requirements.

PATIENT AND PUBLIC INVOLVEMENT
Two study participants and public involvement representatives advised the research group from the inception of the study. They were also members of the steering committee and advised throughout the study.

For peer review only

REFERENCES:

1. Oxford English Dictionary [Internet]. Oxford University Press; c2020. Internet; [cited 17 August 2019]; [about 1 screen]. Available from: https://www.oed.com/viewdictionaryentry/Entry/248411

2. Tan S, Goonawardene N. Internet Health Information Seeking and the Patient-Physician Relationship: A Systematic Review. *JMIR*. 2017;19(1):e9.

3. Prescott, C. Internet access – households and individuals, Great Britain:2020 - Office for National Statistics [Internet]. Ons.gov.uk. 2020 [Accessed 16 March 2021]. Available from:https://www.ons.gov.uk/peoplepopulationandcommunity/householdcharacteristics/homeinternetandsocialmediausage/bulletins/internetaccesshouseholdsandindividuals/2020

4. '3. People will get more control over their own health and more personalised care when they need it. [Internet] longtermplan.nhs.uk 2021. [Accessed 16 March 2021]. Available from: https://www.longtermplan.nhs.uk/online-version/chapter-1-a-new-service-model-for-the-21st-century/3-people-will-get-more-control-over-their-own-health-and-more-personalised-care-when-they-need-it/

5. Department of Health. Self-care – a real choice: self-care support — a practical option. London: HMSO, 2005.

6. '3 before GP': new RCGP mantra to help combat winter pressures in general practice [Internet]. Rcgp.org.uk. 2018 [cited 8 September 2018]. Available from: http://www.rcgp.org.uk/aboutus/news/2017/december/3-before-gp-new-rcgp-mantra-tohelp-combat-winter-pressures-ingeneral-practice.aspx

For peer review only - http://bmjopen.bmj.com/site/about/guidelines.xhtml

7. Dyer C. Doctors should not cherry pick what information to give patients, court rules BMJ 2015;350:h1414

8. "Dr Google enters 80% of my consultations", warns RCGP chair [Internet]. Available from:http://www.pulsetoday.co.uk/news/gp-topics/it/dr-google-enters-80-of-myconsultations-warnsrcgp-chair/20035400.article [Accessed 5 August 2019]

9. Bowes P, Stevenson F, Ahluwalia S and Murray E. 'I need her to be a doctor': patients' experiences of presenting health information from the internet in GP consultations. *Br J of Gen Pract*. 2012;732.

10. Ahluwalia S, Murray E, Stevenson F, Kerr C, Burns J. 'A heartbeat moment': qualitative study of GP views of patients bringing health information from the internet to a consultation. *Br J of Gen Pract*. 2010:60(571):88-94

11. C Bloor MJ, Hororbin GW. Conflict and conflict resolution in doctor/patient interactions. In: Ox C, Mead A, eds. A Sociology of Medical Practice. 1st ed. London: Collier Macmillan London; 1975. p.271-284.

12. Seguin M, Hall L, Atherton H, Barnes R, Leydon G, Murray E et al. Protocol paper for the 'Harnessing resources from the internet to maximise outcomes from GP consultations (HaRI)' study: a mixed qualitative methods study. *BMJ Open*. 2018;8(8):e024188.

13. Ritchie J, Lewis J. Qualitative research practice. Thousand Oaks, Calif.: SAGE; 2003.

14. Department of Health & Social Care, National Information Board. Policy Paper: Personalised health and care 2020: a framework for action. Published 13 November 2014.

15. Braun V, Clarke V. Using thematic analysis in psychology. *Qual Res Psychol.* 2006;3(2):77-101.

16. Nowell L, Norris J, White D, Moules N. Thematic Analysis. *Int J Qual Methods.* 2017;16(1):1-13.

17. Wang J, Ashvetiya T, Quaye E, Parakh X, Martin S S. Online Health Searches and Their Perceived Effects on Patients and Patient-Clinician Relationships: A Systematic Review. A*m J Med.* 2018;131(10).

18. Diviani N, Haukeland Fredriksen E, Meppelink C S, Mullan J, Rich W, Therkildsen Sudmann T. Where else would I look for it? A five-country qualitative study on purposes, strategies, and consequences of online health information seeking. *Journal of Public Health Research.* 2019;8(1): 33-39.

19. Parsons T. The social system. Psychology Press; 1991.

For peer review only - http://bmjopen.bmj.com/site/about/guidelines.xhtml

20. Pilnick A, Dingwall R. On the remarkable persistence of asymmetry in doctor/patient interaction: a critical review. *Soc Sci Med*. 2011;72(8):1374-1382. doi:10.1016/j.socscimed.2011.02.033

21. Heritage J (2012) Epistemic in conversation in Sidnell, J. & Stivers, T. (Eds.) The Handbook of Conversation Analysis. Blackwell-Wiley P 370-394.

For peer review only

SUPPLEMENTAL MATERIAL 1 – PATIENT CONSENT FORM

Title of Project: **Harnessing resources to maximise outcomes from GP consultations (HaRI): a qualitative study**

Please initial all boxes

1. I confirm that I have read and understand the information sheet provided dated 15th July 2016 (v 1.2) for the above study. I have had the opportunity to consider the information, ask questions and have had these answered satisfactorily. ☐

2. I understand that my participation is voluntary and that I am free to withdraw at any time up until the end of data collection at this site without giving any reason. ☐

3. I understand that my interview will be audio-recorded. ☐

4. I understand that the interview will be transcribed by a professional transcribing company with details anonymised to protect my identity. ☐

5. I agree to the use of anonoymised verbatim quotation for the purposes of education and teaching (for example publication, conference presentations). ☐

CONSENT TO POINTS 6 AND 7 IS OPTIONAL AND NOT REQUIRED FOR STUDY PARTICIPATION

6. I consent for the transcript of the interview to be placed into an archive at UCL to allow data to be used, subject to an appropriate protocol and ethical approval, by other researchers. I understand this carries a potential risk that I might be identified. ☐

7. I consent for the audio tape to be placed into an archive at UCL to allow data to be used, subject to an appropriate protocol and ethical approval, by other researchers. I understand this carries a potential risk that I might be identified. ☐

______________________	______________________	______________________
Name of Participant	Date (DD/MM/YY)	Signature
______________________	______________________	______________________
Name of person taking consent	Date (DD/MM/YY)	Signature

For peer review only

SUPPLEMENTAL MATERIAL 2 – INTERVIEW TOPIC GUIDE

Interview Topic Guide: Patients

Topic Guide : Interviews with patients

Harnessing resources from the internet to maximise outcomes from GP consultations (HaRI): a qualitative study

1. Consultation

Could you start by describing what you can remember about the consultation we videorecorded.

2. Sources of information and advice

Before your appointment with [GP's name], did you seek information or advice about symptoms, conditions, treatments or health services?

INTERNET MENTIONED

Focusing on the information/advice you looked for on the internet, can you remember what information/advice you were looking for?

Did you refer to the information/advice you looked for on the internet during your consultation with [GP's name]?

-Yes

How did the doctor respond?

-No

Was there any reason why you did not refer to the information/advice you looked for on the internet during your consultation?

NO MENTION OF INTERNET

Did you consult the internet for information or advice about symptoms, conditions, treatments or health services before your appointment with [GP's name]?

-Yes

Did you refer to this during your consultation?

-Yes

For peer review only

How did the doctor respond?

-No

Was there any reason why you did not refer to the information/advice you looked for on the internet during your consultation?

……………………………………………………………………………………………..

-No

Have you looked for information or advice about symptoms, conditions, treatments or health services on the internet before your consultation with the doctor on some other occasion?

YES

Did you refer to this during your consultation?

-Yes

How did the doctor respond?

-No

Was there any reason why you did not refer to the information or advice you looked for on the internet during your consultation?

3. GP use of internet for diagnosis/treatment recommendation

Has the doctor ever looked something up on the internet during your consultation?

-Condition

-Treatment

YES

-Can you remember any particular occasions?

-What were your thoughts on the doctor doing that?

NO

-What do you think about doctors looking up information on the internet during consultations?

4. GP use of internet for explaining condition/treatment

Has the doctor ever used information from the internet to help him/her explain a condition or treatment?

-Talked through information on a webpage

For peer review only

-Images

**YES**
-Can you remember any particular occasions when your doctor has done this?
-What were your thoughts on the doctor doing that?

**NO**
What do you think about doctors using information from the internet to help explain a condition or treatment during consultations?

5. GP printing out information from the internet for patient to take away

Has the doctor ever printed out information from the internet about a health condition or treatment for you to take away and read?

**YES**
-Can you remember any particular occasions when your doctor has done this?
-What were your thoughts on the doctor doing that?

**NO**
What do you think about doctors printing out information from the internet for patients to take away and read?

6. GP recommendation

Has the doctor ever recommended a particular website during your consultation?

**YES**
-Can you remember any particular occasions when your doctor has done this?
-What were your thoughts on the doctor doing that?

7. GP warning

Has the doctor ever suggested that you don't look for information or advice on the internet?

**YES**
-What did you think about the doctor doing that?

For peer review only

NO

-What do you think about doctors suggesting to patients that they should not look for health information or advice on the internet?

8. Anything to add

Is there anything you would like to add about:

-The information patients and GPs use in and outside of consultations to manage health problems

-GP consultations more generally

9. Reflections on taking part in the research

How did you find participating in the study?

For peer review only

COREQ (COnsolidated criteria for REporting Qualitative research) Checklist

A checklist of items that should be included in reports of qualitative research. You must report the page number in your manuscript where you consider each of the items listed in this checklist. If you have not included this information, either revise your manuscript accordingly before submitting or note N/A.

Topic	Item No.	Guide Questions/Description	Reported on Page No.
**Domain 1: Research team and reflexivity**			
Personal characteristics			
Interviewer/facilitator	1	Which author/s conducted the interview or focus group?	
Credentials	2	What were the researcher's credentials? E.g. PhD, MD	
Occupation	3	What was their occupation at the time of the study?	
Gender	4	Was the researcher male or female?	
Experience and training	5	What experience or training did the researcher have?	
Relationship with participants			
Relationship established	6	Was a relationship established prior to study commencement?	
Participant knowledge of the interviewer	7	What did the participants know about the researcher? e.g. personal goals, reasons for doing the research	
Interviewer characteristics	8	What characteristics were reported about the inter viewer/facilitator? e.g. Bias, assumptions, reasons and interests in the research topic	
**Domain 2: Study design**			
Theoretical framework			
Methodological orientation and Theory	9	What methodological orientation was stated to underpin the study? e.g. grounded theory, discourse analysis, ethnography, phenomenology, content analysis	
Participant selection			
Sampling	10	How were participants selected? e.g. purposive, convenience, consecutive, snowball	
Method of approach	11	How were participants approached? e.g. face-to-face, telephone, mail, email	
Sample size	12	How many participants were in the study?	
Non-participation	13	How many people refused to participate or dropped out? Reasons?	
Setting			
Setting of data collection	14	Where was the data collected? e.g. home, clinic, workplace	
Presence of non-participants	15	Was anyone else present besides the participants and researchers?	
Description of sample	16	What are the important characteristics of the sample? e.g. demographic data, date	
Data collection			
Interview guide	17	Were questions, prompts, guides provided by the authors? Was it pilot tested?	
Repeat interviews	18	Were repeat inter views carried out? If yes, how many?	
Audio/visual recording	19	Did the research use audio or visual recording to collect the data?	
Field notes	20	Were field notes made during and/or after the inter view or focus group?	
Duration	21	What was the duration of the inter views or focus group?	
Data saturation	22	Was data saturation discussed?	
Transcripts returned	23	Were transcripts returned to participants for comment and/or	

For peer review only

Topic	Item No.	Guide Questions/Description	Reported on Page No.
		correction?	
**Domain 3: analysis and findings**			
Data analysis			
Number of data coders	24	How many data coders coded the data?	
Description of the coding tree	25	Did authors provide a description of the coding tree?	
Derivation of themes	26	Were themes identified in advance or derived from the data?	
Software	27	What software, if applicable, was used to manage the data?	
Participant checking	28	Did participants provide feedback on the findings?	
Reporting			
Quotations presented	29	Were participant quotations presented to illustrate the themes/findings? Was each quotation identified? e.g. participant number	
Data and findings consistent	30	Was there consistency between the data presented and the findings?	
Clarity of major themes	31	Were major themes clearly presented in the findings?	
Clarity of minor themes	32	Is there a description of diverse cases or discussion of minor themes?	

Developed from: Tong A, Sainsbury P, Craig J. Consolidated criteria for reporting qualitative research (COREQ): a 32-item checklist for interviews and focus groups. *International Journal for Quality in Health Care*. 2007. Volume 19, Number 6: pp. 349 – 357

Once you have completed this checklist, please save a copy and upload it as part of your submission. DO NOT include this checklist as part of the main manuscript document. It must be uploaded as a separate file.

For peer review only

Standards for Reporting Qualitative Research (SRQR)*

http://www.equator-network.org/reporting-guidelines/srqr/

Title and abstract

	Page/line no(s).
**Title** - Concise description of the nature and topic of the study Identifying the study as qualitative or indicating the approach (e.g., ethnography, grounded theory) or data collection methods (e.g., interview, focus group) is recommended	p.1 / l.2-3
**Abstract** - Summary of key elements of the study using the abstract format of the intended publication; typically includes background, purpose, methods, results, and conclusions	p.3/l.1-21

Introduction

**Problem formulation** - Description and significance of the problem/phenomenon studied; review of relevant theory and empirical work; problem statement	p.3/l.32-p.4/l.13
**Purpose or research question** - Purpose of the study and specific objectives or questions	p.4/l.15-19

Methods

**Qualitative approach and research paradigm** - Qualitative approach (e.g., ethnography, grounded theory, case study, phenomenology, narrative research) and guiding theory if appropriate; identifying the research paradigm (e.g., postpositivist, constructivist/ interpretivist) is also recommended; rationale**	p.4/l.23-32
**Researcher characteristics and reflexivity** - Researchers' characteristics that may influence the research, including personal attributes, qualifications/experience, relationship with participants, assumptions, and/or presuppositions; potential or actual interaction between researchers' characteristics and the research questions, approach, methods, results, and/or transferability	p.5/l.12-13
**Context** - Setting/site and salient contextual factors; rationale**	p.4/l.27-29
**Sampling strategy** - How and why research participants, documents, or events were selected; criteria for deciding when no further sampling was necessary (e.g., sampling saturation); rationale**	p.4/l.41- p.5/l.11
**Ethical issues pertaining to human subjects** - Documentation of approval by an appropriate ethics review board and participant consent, or explanation for lack thereof; other confidentiality and data security issues	p.5/l.5-11
**Data collection methods** - Types of data collected; details of data collection procedures including (as appropriate) start and stop dates of data collection and analysis, iterative process, triangulation of sources/methods, and modification of procedures in response to evolving study findings; rationale**	p.4/l.24-32 & p.5/l.13-15

For peer review only - http://bmjopen.bmj.com/site/about/guidelines.xhtml

For peer review only

**Data collection instruments and technologies** - Description of instruments (e.g., interview guides, questionnaires) and devices (e.g., audio recorders) used for data collection; if/how the instrument(s) changed over the course of the study	p.5/l.15-17
**Units of study** - Number and relevant characteristics of participants, documents, or events included in the study; level of participation (could be reported in results)	p.4/l.35
**Data processing** - Methods for processing data prior to and during analysis, including transcription, data entry, data management and security, verification of data integrity, data coding, and anonymization/de-identification of excerpts	p.5/l.17-19
**Data analysis** - Process by which inferences, themes, etc., were identified and developed, including the researchers involved in data analysis; usually references a specific paradigm or approach; rationale**	p.5/l.22-27
**Techniques to enhance trustworthiness** - Techniques to enhance trustworthiness and credibility of data analysis (e.g., member checking, audit trail, triangulation); rationale**	p.5/l.27-30

Results/findings

**Synthesis and interpretation** - Main findings (e.g., interpretations, inferences, and themes); might include development of a theory or model, or integration with prior research or theory	p.6/l.2-8<br><br>p.7/l.7-11 & l.21-23 & l.31-32 l.38-41<br><br>p.8/l.11-12 & l.16-18 & l.24-27 & l.37-39<br><br>p.9/l.1-3 & l.9-13
**Links to empirical data** - Evidence (e.g., quotes, field notes, text excerpts, photographs) to substantiate analytic findings	p.7/l.1-5 & l.13-19 & l.25-29 & l.34-36<br><br>p.8/l.1-9 & l.14 & l.20-22 & l.29-35 & l.41-43<br><br>p.9/ l.5-7 & l.15-17

Discussion

**Integration with prior work, implications, transferability, and contribution(s) to the field -** Short summary of main findings; explanation of how findings and conclusions connect to, support, elaborate on, or challenge conclusions of earlier scholarship; discussion of scope of application/generalizability; identification of unique contribution(s) to scholarship in a discipline or field	p.9/l.21-26<br>p.9/l.36 -<br>p.10/l.15<br>p.10/l.19-25
**Limitations** - Trustworthiness and limitations of findings	p.9/l.29-43

For peer review only - http://bmjopen.bmj.com/site/about/guidelines.xhtml

For peer review only

Other

**Conflicts of interest** - Potential sources of influence or perceived influence on study conduct and conclusions; how these were managed	p.10/l.40
**Funding** - Sources of funding and other support; role of funders in data collection, interpretation, and reporting	p.10/l.34-38

*The authors created the SRQR by searching the literature to identify guidelines, reporting standards, and critical appraisal criteria for qualitative research; reviewing the reference lists of retrieved sources; and contacting experts to gain feedback. The SRQR aims to improve the transparency of all aspects of qualitative research by providing clear standards for reporting qualitative research.

**The rationale should briefly discuss the justification for choosing that theory, approach, method, or technique rather than other options available, the assumptions and limitations implicit in those choices, and how those choices influence study conclusions and transferability. As appropriate, the rationale for several items might be discussed together.

Reference:

O'Brien BC, Harris IB, Beckman TJ, Reed DA, Cook DA. **Standards for reporting qualitative research: a synthesis of recommendations.** *Academic Medicine*, Vol. 89, No. 9 / Sept 2014 DOI: 10.1097/ACM.0000000000000388

For peer review only
